# The Supporting Role of Mentees’ Peers in Online Mentoring: A Longitudinal Social Network Analysis of Peer Influence

**DOI:** 10.3389/fpsyg.2020.01929

**Published:** 2020-08-14

**Authors:** Manuel D. S. Hopp, Heidrun Stoeger, Albert Ziegler

**Affiliations:** ^1^Educational Psychology and Research on Excellence, University of Erlangen–Nuremberg, Nuremberg, Germany; ^2^School Research, School Development, and Evaluation, University of Regensburg, Regensburg, Germany

**Keywords:** online mentoring, peer influence, social network analysis, RSiena, STEM

## Abstract

Studies show that online mentoring is an effective measure to support girls in STEM (science, technology, engineering, and mathematics), especially if it also allows for networking with other participants on the mentoring platform. However, research is missing on peer influence. This topic seems especially crucial in programs for adolescents as peer influence plays an important role at this age. In our study, we investigated peer influence on mentoring outcomes – confidence in own STEM abilities and STEM-related activities – in an online mentoring program in STEM for secondary school girls (*N* = 124, *M* = 14.3 years, SD = 2.2 years, age range: 11–19 years). The program provides girls with at least 1 year of one-on-one interaction with a personal female mentor who has a college degree in a STEM subject. Participants can also interact with other participants on the platform. We used a longitudinal social network analysis approach to examine peer influence on mentoring outcomes. Our results indicate that both mentoring outcomes – mentees’ confidence in own STEM abilities and STEM-related activities – are influenced by peers moderated by the mentees’ own age. Younger mentees tended to become more similar to their peers regarding confidence in own STEM abilities and STEM-related activities, whereas older mentees tended to become more dissimilar over time. In addition, peer group size had a positive effect on confidence in own STEM abilities, but not on STEM-related activities. This effect was moderated by the mentee’s age. Overall, peers have a positive influence on the measured mentoring outcomes, especially for young mentees.

## Introduction

In Germany and many other industrial countries, the participation rate of females in STEM (science, technology, engineering and mathematics) is deficient, especially in engineering and computer-science (Statistisches Bundesamt, 2018). For example, only about one in five STEM academics, and about one out of nine STEM skilled labors is female. There are several external reasons why this discrepancy occurs. Some argue that one possible reason for this decline relates to negative stereotypes about STEM fields (e.g., Kessels et al., 2006), such as the stereotype that women working in the STEM field are unfeminine (Yoder and Schleicher, 1996; Smeding, 2012). These pervasive stereotypes negatively impact attitudes toward STEM, competence beliefs, and career preferences for females (e.g., Steffens et al., 2010; Nosek and Smyth, 2011; Cundiff et al., 2013). This gender discrepancy starts early. Studies have demonstrated that students’ interest in STEM subjects decreases throughout their school careers (Frenzel et al., 2010), indicating that interventions aimed at encouraging STEM involvement should begin during school years while students are still forming their decisions.

An effective measure to change the situation is mentoring (e.g., McCord et al., 2009; Stoeger et al., 2013). It combines various advantages. For example, mentors can answer mentees’ questions about STEM, discuss interesting STEM topics and work on STEM projects with their mentees, all of which have a positive influence on mentees’ self-confidence, STEM-related activities, and STEM interest (Harsh et al., 2011; Schultz et al., 2011). That female mentors also act as role models plays an especially important role when it comes to supporting girls in STEM (Eccles, 1984). Online mentoring, too, is notably advantageous (Stoeger et al., 2013, 2016). Because of its time and local flexibility, online mentoring enables to include extensive numbers of female mentors and mentees (who due to the low participation rates in STEM cannot be easily found for offline programs) – and thereby offers networking opportunities with both female high-status role models (mentors) as well as female peer role models (mentees). Studies outside the field of mentoring indicate that same-age role models are particularly effective in changing the perception of STEM fields as unfeminine (Kessels et al., 2014) and can act as social vaccines who inoculate girls against negative influences on their STEM self-concepts (Dasgupta, 2011; Stout et al., 2011; Dennehy and Dasgupta, 2017). Research within the field of mentoring shows that online-mentoring that combines one-on-one mentoring with networking with same-age role models is more effective than one-on-one mentoring (Stoeger et al., 2017). However, to the best of our knowledge there is no research on peer influences in the context of online mentoring for girls in STEM, which is unfortunate as especially during adolescence, peer influence becomes particularly important (DeLay et al., 2016).

The main objective of this study is to investigate whether mentoring outcomes (namely confidence in own STEM abilities and STEM-related activities) of mentees are influenced by networking with other mentees (peer influence). As research shows that peer influence differs during development (e.g., Steinberg and Monahan, 2007), we also investigate the moderating role of age when it comes to peer influence on mentoring outcomes. Furthermore, we consider that the contributing role of the size of a mentee’s peer group (as indicated by e.g., Wang et al., 2016) might impact mentoring outcomes.

### Online Mentoring for Girls in STEM

Girls show a lower interest in STEM compared to boys and report lower self-confidence in their own STEM abilities (Hoffmann, 2002; Litzler et al., 2014; Ellis et al., 2016; e.g., Cheryan et al., 2017; Hand et al., 2017), which in turn can lead to reduced involvement in STEM-related activities (e.g., Shrauger and Schohn, 1995). Mentoring offers a good opportunity for extracurricular intervention. Mentoring is commonly defined as a relatively stable dyadic relationship between an experienced mentor and his or her less experienced mentee. It is characterized by mutual trust and goodwill, and it aims to promote learning and development as well as the mentee’s progress (Ziegler, 2009). In mentoring programs for girls in STEM, most often female, higher status, and older role models act as mentors (Pleiss and Feldhusen, 1995; Khare et al., 2013; e.g., Dawson et al., 2015). In online mentoring programs for girls in STEM, one-on-one mentoring sometimes is complemented by networking opportunities with other mentees and mentors that are similarly interested in STEM (Stoeger et al., 2016, 2017). Research shows that these networking opportunities can lead to better outcomes than one-on-one mentoring alone (Stoeger et al., 2016, 2017). One can only speculate about the reasons for the higher effectiveness of a combination of one-on-one mentoring and networking opportunities in online mentoring for girls in STEM. First, the increased number of communication partners seems to lead to more STEM communication and more STEM-related activities (Stoeger et al., 2016, 2017). Second, the specific influence of peers might also play a role. In addition to female higher status role models, programs of this kind also offer peers as role models, which seems to have an especially big influence on the development of STEM self-concept (Dasgupta, 2011) and STEM elective behavior in female students (Dasgupta and Stout, 2014). So far, there is little research on peer influence in online mentoring in STEM.

### Peer Influence in Online Mentoring

Due to the relatively low interest of girls in STEM and its continual decrease during school time (Gardner, 1985; Hoffmann et al., 1985; Kerr and Robinson Kurpius, 2004), it is difficult for girls interested in STEM to find female peers with a similar STEM interest. This is problematic because peers act as role models and as a comparison level when it comes to abilities and behavior (Schunk, 1987, 1989). In contexts where objective standards of behavior are unclear or unavailable – as is the case for STEM subjects for girls due to missing female role models – peers are better role models than grownups; most effective are peers who have a similar or slightly higher experience or status (Schunk, 1987). Same-age female role models are particularly effective in changing the perception of STEM fields as unfeminine (Kessels et al., 2014) and peer support plays an important role in girls’ willingness to persist in STEM (Schoon and Eccles, 2014). Peer networks offer opportunities for interaction, observation of others, and facilitate access to activities (Dweck and Goetz, 1978). Furthermore, peers can positively influence self-efficacy (especially when less pronounced, see Schunk, 1989), which seems to be important for girls in STEM as they often do not dare to choose STEM, even if their achievements are high (Eccles, 1994). Peer mentoring makes use of the positive influence that peers can have on a wide range of mentoring objectives (Colvin and Ashman, 2010; Karcher, 2013).

Through online mentoring, girls interested in STEM can get access to a social environment where their STEM interest is valued, and they can meet (sometimes for the first time) a large number of other girls and women who are similarly interested in STEM. Social learning theories (Zimbardo and Leippe, 1991) suggest that this change in the social environment – especially through peer influence – can lead to changes in their own behavior and values (i.e., mentoring outcomes). However, most research examines peer influence in offline contexts, e.g., the classroom, where students physically interact and see the actions of each other. In these contexts, it could be demonstrated that peers influence each other in various ways (Steinberg and Silverberg, 1986; prosocial behavior, Wentzel et al., 2004; smoking, Mercken et al., 2010; e.g., delinquent behavior, Kerr et al., 2012). For some time, it was doubted that a similar influence can be found in online contexts. It was assumed that due to missing physical contact and therefore missing facial expressions and gesture in computer-based communication, emotional and observational information gets lost that is important for peer influence. However, it is now known that with the help of Emoji usage and image-based communication, a proxy for “real” interaction can be achieved (e.g., Kralj Novak et al., 2015). Indeed, several studies from different fields have shown evidence of peer influence in online (social) networks (Hui and Buchegger, 2009; Aral and Walker, 2011, 2012; Lewis et al., 2012; Huang et al., 2014; Bapna and Umyarov, 2015). Moreover, the current state of research shows clear indications of moderation of age on peer influence (in online settings). In addition, peer network size plays a role in peer influence. This will be discussed in more detail in the next two sections.

#### Age as a Moderator of Peer Influence

Peer influence seems to be important to differing degrees in various stages of development (Brechwald and Prinstein, 2011). Studies have demonstrated that individuals appear more likely to be influenced by their peers in earlier developmental stages of adolescence than in later stages (e.g., Steinberg and Monahan, 2007; Aral and Walker, 2012). Again, most studies have been conducted in offline contexts. For example in a sample of over 3600 individuals ranging in age from 10 to 30 years, a negative linear relationship between age and peer influence was found (Steinberg and Monahan, 2007). Peer influence decreased steadily, particularly in individuals aged between 14 and 18 years. Similar results have also been found in earlier works by Steinberg et al. (1997). These results are consistent with the effects of “cross-mentoring,” in which older adolescents act as mentors for younger teenagers. For example, cross-mentoring can help develop self−esteem, social skills, and behavioral competence in mentees (Karcher, 2005).

While so far, no research exists on age effects of peer influence in online mentoring, studies in online contexts suggest similar results for this area. For example, Aral and Walker (2012) showed that younger users were more susceptible to peer influence than older ones when it came to Facebook applications. Bapna and Umyarov (2015) also found that younger Facebook users were more influenced by their Facebook peers than older ones.

#### Peer Group Size as a Contributing Factor

Another aspect that might contribute to the peer influence on mentoring outcomes in online mentoring is the size of the online peer group mentees interact with on the mentoring platform. On online mentoring platforms, a mentee can potentially interact (via messaging tools) with a varying number of other mentees. Research from offline context indicates that the larger the peer group an individual interacts with, the more pronounced the peer influence (e.g., Wang et al., 2016). An explanation for this might be a kind of “contagiousness.” Similar to disease infections – where it is evident that the more people an individual has contact with, the more likely he or she will become infected with a contagious disease (Ferrari et al., 2006) – interacting with a large number of peers with similar values, interests, and behaviors is more likely to lead to an adaption of the same values, interests, and behaviors. In the offline context, there is ample evidence of “contagiousness” for various attributes, values, and behaviors (obesity, Christakis and Fowler, 2007; e.g., delinquent behavior, Burk et al., 2008; Dijkstra et al., 2010; smoking, Mercken et al., 2010; depression, Schaefer et al., 2011; tastes in books, films, and movies, Lewis et al., 2012). For the online mentoring context, this might translate to: the more conversational partners a mentee has on the mentoring platform, the more likely the mentee will be influenced by his or her peers and thus, adopt their level of confidence in own STEM abilities or STEM-related activities.

### The Present Study

There is ample evidence that online mentoring is an effective measure in the support of girls in STEM. An advantage of online mentoring programs is that girls not only profit from an individual mentor – in many cases an adult, high status, female role model – but that they can also interact with many other girls (and women) interested in STEM. Although there is a lack of research on peer influence in the context of online mentoring, based on research on peer influence from offline contexts, as well as from online contexts unrelated to mentoring, we expect that peer influence affects mentoring outcomes. Research from the offline context shows that peers influence (STEM-related) self-concept (Schunk, 1989; Dasgupta, 2011) and activities (Dasgupta and Stout, 2014). Thus, our first hypothesis is:

*Hypothesis 1*: Mentees are subject to peer influence in online mentoring, regarding (H1a) confidence in own STEM abilities and (H1b) STEM-related activities.

We test this hypothesis in the following way: If peer influence is present, a random mentee’s value of confidence in own STEM abilities and STEM-related activities over time should approach the (average) value of her peer group. To obtain evidence for this hypothesis and the following hypotheses, we used a longitudinal social network analysis approach. Here, the two main elements are the mentees and their peer relationships. In the method we applied, the evolution of the peer relationship network as well as its influence on the mentoring outcomes are considered simultaneously. The driving effects of this co-evolution are represented by log-odds values. A more detailed introduction to network analysis and the method used herein can be found in section “A Primer of Longitudinal Social Network Analysis.” A positive, significant value of the corresponding peer influence effect would support hypothesis 1.

Research suggests that peer influence effects are moderated by age. Both studies from the offline context (Steinberg and Monahan, 2007; Aral and Walker, 2012) as well as from the online context – unrelated to online mentoring (Aral and Walker, 2012; Bapna and Umyarov, 2015) – suggest that younger individuals are more likely to be influenced by their peers than older individuals. Thus, our second hypothesis is:

*Hypothesis 2*: The mentee’s age moderates the peer influence that a mentee is subject to in the following way: younger mentees are more susceptible to peer influence (than older mentees) in both (H2a) their confidence in STEM abilities and (H2b) their STEM-related activities.

To test hypothesis 2, we included a moderation term of age on the respective peer influence effect. A negative, significant value of the corresponding effect would support our hypothesis.

Another aspect that might contribute to mentoring success is the size of the peer group. The role of peer group size has been demonstrated for various attributes, values, and behaviors in the offline context (Christakis and Fowler, 2007; Burk et al., 2008; Mercken et al., 2010; Schaefer et al., 2011; Lewis et al., 2012; Wang et al., 2016). Based on these findings, we assume that the larger the online peer group of a mentee (i.e., the number of peer mentees an individual interacts with on the online mentoring platform), the more positive the mentoring outcomes. Thus, our third hypothesis is:

*Hypothesis 3*: The size of the peer group (operationalized by the number of peers that regularly send messages to the mentee) contributes positively to the mentoring outcomes in the following way: The larger the size of the peer group a mentee interacts with, the more positive the development of her (H3a) confidence in own STEM abilities and (H3b) STEM-related activities.

A significantly positive value of the corresponding peer group size effect would support hypothesis 3. Similar to the influence of the peers on mentoring outcomes, the influence of the peer group size on the mentoring outcomes might also be moderated by the age of the specific mentee (Steinberg and Monahan, 2007; Brechwald and Prinstein, 2011). Thus, our fourth hypothesis is:

*Hypothesis 4:* The influence of the size of the peer group (operationalized by the number of peers that regularly send messages to the mentee) on the mentoring outcomes is moderated by the mentee’s age in the following way: The older mentees are, the less they benefit from an increasing size of their peer group regarding (H4a) their confidence in STEM abilities and (H4b) their STEM-related activities.

To address hypothesis 4, we included a moderation term of age on the peer group size effect of hypothesis 3. In our applied method, a negative, significant value of the corresponding effect would support hypothesis 4.

## Materials and Methods

### A Primer of Longitudinal Social Network Analysis

The communication data between mentees and mentors in online mentoring (e.g., emails and chat messages) can be used to create social networks. In these networks, mentees and mentors are called nodes or vertices, the communication paths between the persons are called ties or edges. If, for example, two mentees exchange messages with each other, a new edge is created between these two nodes. During the mentoring process, new edges are created, and old ones are dissolved. Because of these networking activities (creation of new edges and dissolvement of existing edges) the individual mentees do not develop independently of each other, which leads to (statistical) interdependence in the sample. This dependency contrasts with the assumption of the independence of many statistical methods (e.g., linear regression). Modeling and analyzing changing networks and mentee attributes (i.e., mentoring outcomes) require alternative statistical methods. For this reason, we analyzed our data with the help of stochastic actor-oriented modeling, implemented in the R package RSiena (R Core Team, 2017; Ripley et al., 2018). Modeling peer influence without taking the changing network structure into account can lead to incorrect results (Aral et al., 2009; Steglich et al., 2010; Shalizi and Thomas, 2011). Thus, longitudinal network analyses (in our case a stochastic actor oriented model) are used to get a clearer idea about the co-evolution of measured attributes (in our case mentees’ confidence in own STEM abilities and STEM-related activities) and social networks (in our case peer networks). In this context, two main driving forces must be differentiated: *selection* and *influence*.

#### Selection and Influence

The term *selection* describes that individuals consciously (de-)select their peers based on certain criteria, in many cases – especially during adolescence – based on similarity concerning demographic attributes, such as age, gender and ethnicity (e.g., Kupersmidt et al., 1995). In online mentoring for STEM interested girls, for example, similar age is expected to be one driving factor for the creation of new edges between mentees. This effect is also known under the term homophily and is not only restricted to demographic variables, but also includes non-demographic attributes like attending the same school class (McPherson et al., 2001). In our online mentoring context, a non-demographic attribute would be the affiliation to “mentoring groups” of two mentors and two mentees on the platform (for a detailed description refer to section “Measures”).

The term *influence* describes the effect that peers can have on certain attributes of an individual (in our case mentoring outcomes). This means that the peer network mentees interact with might affect their mentoring outcomes (influence).

It is important to mention that within social networks (and in our case the peer networks of mentees) selection and influence processes take place simultaneously and are interwoven (Steglich et al., 2010; Shalizi and Thomas, 2011). Thus, for a better understanding of the processes, it is necessary to disentangle influence from selection.

#### Disentangling Selection From Influence

Network (and other) data are usually not collected continuously, but in (one or more) snapshots, often referred to as “waves.” When we examine a snapshot of a social network and look at one dyad of peer-mentees (i.e., the smallest social group of two connected mentees), the following problem can arise: If two peer-mentees share the same behavior or attribute, we cannot tell whether this similarity arose because of (1) *selection* or (2) *influence* (provided that this behavior or attribute is changeable).

(1) Similarity arose due to *selection* if mentee A and mentee B already shared the same behavior or attribute (in our case confidence in own STEM abilities and STEM-related activities) before the start of their peer relationship and chose to interact with each other as peers because of this similarity. (2) Similarity arose due to *influence* if mentees A and B shared a different expression in a behavior or attribute (in our case confidence in own STEM abilities and STEM-related activities) before forming their peer relationship. However, through the established peer relationship one mentee’s behavior or attribute spread to her peer and therefore changed her attribute. Note that in this example, we only speak of “positive influence,” i.e., an influence in which both participants end up with the more similar behavior or attributes. Opposing effects can also occur so that the behavior or attributes become more dissimilar.

Thus, in order to examine peer influence regarding our mentoring outcome variables (i.e., confidence in own STEM abilities and STEM-related activities), it is necessary to simultaneously consider whether these variables affect the development of the peer relationship network (i.e., whether, for example, mentees with high confidence tend to establish more peer relationships than mentees with low confidence). RSiena addresses the mentioned issues and some further peculiarities of longitudinal network data (Snijders, 2001, 2017; Snijders et al., 2010; Ripley et al., 2018).

#### Main Data Analysis Method: RSiena – A Stochastic Actor-Oriented Model

RSiena stands for the R-package Simulation Investigation for Empirical Network Analysis (Ripley et al., 2018). This stochastic actor-oriented model considers the interplay between selection and influence, and controls for other confounding variables. In the RSiena model, both mentees’ peer relationships and mentoring outcome variables are assumed to change continuously between (the two) measurement points (T1 and T2). Those changes are decomposed into small sequential steps, so-called *ministeps*, in which mentees can change their peer relationships or their respective mentoring outcome variable (Snijders et al., 2010; Ripley et al., 2018). With these ministeps, the goal is to simulate the unobserved changes of the peer relationship network (including all attributes of the mentees) from the first measurement time (i.e., beginning of mentoring) to the second measurement time (i.e., after half a year of mentoring). In each ministep of the simulation, a random mentee is given a “decision opportunity” where she probabilistically changes either her peer relationship or her mentoring outcome variable (i.e., confidence in own STEM abilities or STEM-related activities) according to the mentees’ “preferences.” These preferences are expressed via log-odd ratios of different effects, similar to log-odds of logistic regression (Ripley et al., 2018). For example, if (in the simulation process) mentees tend to establish contact with other mentees who have a similar age to their own, this is expressed in the “similarity in age preference” by a *positive* log-odds value. If, on the other hand, (in the simulation process) mentees tend to establish peer relationships with both younger and older – but not similarly aged – mentees, then the corresponding “similarity in age preference” has a *negative* log-odds value.

Please note: during the simulation process, the mentees (i.e., the actors in the simulation) develop the structure of their peer relationship network; the network also influences the mentoring outcomes of the mentees (e.g., through peer influence). This suggests a causal association. However, as Ripley et al. (2018) wrote, “it does not necessarily reflect a commitment to or belief in any particular theory of action elaborated in the scientific disciplines” (p. 10). In addition, they note that although indications of causal effects may be inferred (as in comparable longitudinal study designs), these must be confirmed by further analyses. This means that the partly causal language used in our result and discussion sections explicitly refers to the simulation process and therefore must not be misinterpreted as actual decisions or beliefs of the mentees! For more information, refer to Snijders et al. (2010).

The model construction in RSiena is based on two categories of effects: effects that determine the development of the network (e.g., similarity in age) and effects that determine the development of attributes (e.g., peer influence regarding confidence in own STEM abilities and STEM-related activities) during the simulation process. A description of all effects that directly determine the development of the mentoring outcomes can be found in Table 1, these include the effects that test our hypotheses, covariates, and network model-specific effects. Moreover, a description of all other effects that solely determine the development of the peer relationship network can be found in the Supplementary Table A1.

**TABLE 1:** Short description of included effects for the simulation of mentoring outcomes in our RSiena models.

Effects	Short description
**Hypotheses related effects**	
*Peer influence (Average similarity) (H1)*	Test for *Hypothesis 1*. Is there peer influence on the mentoring outcomes? The average similarity effect expresses the preference of a mentee to become similar concerning the mentoring outcomes (i.e., concerning confidence in own STEM abilities and STEM-related activities) to the average value of her peers. If it is positive, then mentees tend to get more similar to their peer group, indicating peer influence, if negative, then mentees tend to get dissimilar to their peer group.
*Moderation of mentee’s age on peer influence (Average similarity* × *ego’s age) (H2)*	Test for *Hypothesis 2*. Is there a moderation of mentee’s age on peer influence on mentoring outcomes? The age is centered by RSiena, hence the effect are to be interpreted as follows: A negative value would mean that younger mentees (i.e., beneath the mean age) get more similar to their peers than older mentees (i.e., above the mean age). A positive value would indicate the opposite.
*Peer group size (Indegree) (H3)*	Test for *Hypothesis 3*. Does the peer group size contribute to the peer influence on the mentoring outcomes? The peer group size counts the number of mentees an individual mentee has peer relationships with (i.e., indegree centrality). When it is positive, then it means the bigger the peer group size is, the more likely the mentees mentoring outcomes are influenced positively by the peer group.
*Moderation of mentee’s age on influence of peer group size (Age* × *Indegree) (H4)*	Test for *Hypothesis 4*. Is the influence of the peer group size to the mentoring outcomes moderated by the mentee’s age? The age is centered by RSiena, hence the effect to interpret as follow: A negative value would mean that for each additional peer mentee a younger mentee (i.e., beneath the mean age) is more likely to increase her own mentoring outcome level and an older mentee (i.e., above the mean age) is more likely to decrease her own mentoring outcome level. A positive value would mean the opposite.
**Mentoring outcome related effects**	
*Linear shape*	The linear shape effect expresses the basic drive toward high values on the mentoring outcome. A positive value indicates an increase, and a negative value a decrease.
*Quadratic shape*	The quadratic shape effect is the interaction of the mentoring outcome on itself over time: if it is positive, then it means there is positive feedback, thus the mentoring outcome tends to self-reinforce. In other words: mentees with a high mentoring outcome value at T1 tend to get even higher values at T2 and mentees with a low value at T1 tend to get even lower values at T2. If it is negative, then it can be regarded as negative feedback or a self-correcting mechanism. A mentee with a high value at T1, tend to decrease to T2, and mentees with low values, tend to increase, respectively.
*Experience*	Experience indicates whether a mentee has already participated in the mentoring program before and controls for that
*Age*	The age of the mentee was included as a control variable, in order to estimate the interaction of age with peer influence correctly.
*Mentor relationships*	The number of mentors, a mentee exchanges emails and private chat messages with (operationalized with the same cut-offs used to determine peer relationships) might be a confounding factor of peer influence.

### The Online Mentoring Program CyberMentor as a Study Setting

We investigated our hypotheses with data from the online mentoring program, CyberMentor. CyberMentor is the biggest research-based, online mentoring program in STEM in Germany. It aims to increase the participation rate of female students in STEM. Participants are female students from university preparatory secondary schools throughout Germany, aged 12–18 years. Each girl receives guidance from a personal mentor who has a university degree in STEM. The communication with the mentor as well as with the other mentees and mentors who participate in the program (up to 800 per year) takes place on a secure online platform via internal email, chat and forum systems. Every participant has the possibility to communicate with every other participant. Moreover, each mentoring dyad is linked to another dyad on the platform. This group of four (constituted by two mentees and two mentors) is called a mentoring group. Each mentoring group shares a “virtual room” that contains profile pictures and descriptions of the four participants and gives them direct access to email and private chat messages within the mentoring group. Moreover, each mentoring group has access to a private forum and can initiate a group chat. These measures are intended to increase the communication between the members of the group of four participants. As mentioned above, the general forum, chat and email system also enables communication with other participants outside one’s own mentoring group.

#### Procedure and Sample

Our data collection took place on the online mentoring program CyberMentor. We collected two types of directed data to construct the peer relationship network: emails and private chat messages that participants wrote to each other. We did not include undirected data (i.e., from forums and group chats) in the network construction, as it is not possible to clearly identify who is addressing whom. During the data collection, a total of 430 mentees were registered in CyberMentor. All mentees (all female, *M*_*age*_ = 14.3 years, SD_*age*_ = 2.1, age range: 11–19, with one outlier of 8 years) were enrolled in high achiever-track secondary education in Germany. Their places of residence were scattered all over Germany, which means that mentees did not know each other at the beginning of their mentoring (in any case, the probability of this happening is negligible).

In order to model the peer networks correctly, we only included “active” mentees (*N* = 124; 28.8%) in our sample. We defined “active” mentees as mentees that had at least one peer relationship during mentoring (i.e., at least four written emails or 15 written private chat messages; for more information refer to section “Derived Variables From Log Files”)^1^. To address possible influence of mentors, we also included the number of mentors that a mentee repeatedly wrote emails or private chat messages to during 6 months of mentoring. Here we define a mentor relationship by the same cut-off value as a peer relationship (i.e., at least four written emails or 15 written private chat messages; for more information refer to section “Derived Variables From Log Files”). After 1 year of mentoring, mentees (and mentors) are given the opportunity to participate again. The re-enrolled (referred to as “experienced”) mentees can choose if they want to stay with their former mentor or be assigned to a new mentor. Of the 124 mentees, 100 (80.6%) were first-time participants, and 24 (19.4%) were experienced mentees (i.e., had already participated in CyberMentor at least once before as a mentee).

### Measures

#### Data Collection Process

##### Online questionnaires

All mentees were asked to complete an online questionnaire about confidence in their own STEM abilities and their STEM-related activities before the mentoring year (T1) and after 6 months of mentoring (T2).

##### Log files

Between T1 and T2, program participants’ platform communication was retained via anonymized log files. The data was collected in the following way. An automated script extracted the following attributes of all email and private chat message log files: sender-ID, receiver-ID, and timecode written.

##### Further data

Additional to the questionnaire and log file data, we extracted the mentee’s age at the beginning of the mentoring year, their mentoring group affiliation, and whether they had participated in the program the previous year.

#### Derived Variables From Questionnaires

##### Confidence in own STEM abilities

We assessed students’ confidence in their own STEM abilities using a domain-specific version of the scale “Belief in one’s own abilities.” (Dweck, 1999). This four-item scale measures how confident students are in their (in this case STEM-related) abilities. Two endpoints are formulated as statements, e.g., “I do not have a great deal of confidence in my STEM abilities” vs. “I am confident in my STEM abilities.” Each of the statements in an item pair represents one pole on a six-point scale. A low value represents little confidence in one’s own STEM abilities. Confidence in one’s own STEM abilities was measured both at the beginning of the mentoring (T1) and 6 months later (T2). The scale showed a good one-dimensionality, indicated by McDonald’s ω_*h,T*__1_ = 0.83 and ω_*h,T*__2_ = 0.78 which gives the proportion of variance in scale scores accounted for by a general factor (McDonald, 1999). High ω total values of McDonald’s ω_*t,T*__1_ = 0.88 and ω_*t,T*__2_ = 0.88, respectively indicated a reliable multidimensional composite (Watkins, 2017). The scale showed good internal consistency of Cronbach’s α_*T*__1_ = 0.85 and α_*T*__2_ = 0.83, respectively.

##### STEM-related activities

We used a 9-item scale for assessing mentees’ STEM-related activities (Stoeger et al., 2013). Respondents indicated on a 6-point Likert-type scale (with “1” = strongly disagree and “6” = strongly agree) to what extent they partake in STEM-related activities, e.g., reading STEM-related books or attending STEM-related extracurricular lectures. Sample item: “I very often read articles about STEM topics.” STEM-related activities were measured both at the beginning of mentoring (T1) and 6 months later (T2). The scale showed an acceptable one-dimensionality, indicated by McDonald’s ω_*h,T*__1_ = 0.58 and ω_*h,T*__2_ = 0.59 which gives the proportion of variance in scale scores accounted for by a general factor (McDonald, 1999). High ω total values of McDonald’s ω_*t,T*__1_ = 0.85 and ω_*t,T*__2_ = 0.84, respectively indicated a reliable multidimensional composite (Watkins, 2017). The scale showed good internal consistency of Cronbach’s α_*T*__1_ = 0.81 and α_*T*__2_ = 0.78, respectively.

##### Conversion of questionnaire data

As the method RSiena used for our network analyses needs whole number (integer) values of data, we converted the 1–6 valued decimal number format scales of the two variables with the range of 5 by multiplying all values by 2 and rounding the results to integers, resulting in a 2–12 valued integer number format scale with the range of 10.

#### Derived Variables From Log Files

##### Peer relationship networks

Every mentee of our sample can theoretically have up to 123 peer relationships (with other mentees). These peer relationships are coded in so-called adjacency matrices. In our case, such an adjacency matrix is 124 × 124 in size, i.e., it consists of 15,376 elements. Each line of the matrix codes the relationships of one mentee, whereby the value “1” stands for a peer relationship and the value “0” for no peer relationship. Please note that the method we use does not support weighted relationships, i.e., values higher than “1”). For our longitudinal network analyses, we created two adjacency matrices. One maps all peer relationships for the first 4 weeks, the other maps all peer relationships for the following 5 months of mentoring (see section “Plan of Analysis” for more details). These peer relationships are (in our case) directed. This means a mentee A can have a peer relationship to mentee B, but mentee B does not need to have a peer relationship to mentee A (i.e., reciprocal peer relationships are not required).

To derive the peer relationship networks of the sampled mentees on the online mentoring platform CyberMentor, a proper measure must be set to distinguish when a “real” peer relationship between relevant participants can and cannot be assumed. For a peer relationship between two mentees to be considered as such, repeated communication has to be observed (Roberts and Dunbar, 2011), especially if the communication takes place exclusively on an online platform (Arnaboldi et al., 2013). Previous research suggested several different methods to distinguish strong from weak relationships (Schaefer et al., 2010; Daniel et al., 2013). The underlying assumption of these methods is that all participants in the network know each other. However, this does not apply to the online mentoring context at hand. Thus, we defined an existing (directed) relationship (“1”) from mentee A to another mentee B for a given time (after 4 weeks or 6 months of mentoring), when the number of written messages from mentee A to mentee B laid in the upper quartile of all mentee-to-mentee written messages, i.e., at least four written emails or at least 15 written private chat messages. For example, if mentee A wrote mentee B four emails and seven private chat messages and mentee A wrote mentee C one email and 20 private chat messages in the first 4 weeks, then mentee A had a peer relationship to mentee B as well as to mentee C in the first adjacency matrix (T1).

##### Peer group size

The peer group size is the number of mentees that a specific mentee has peer relationships with. This is identical to the indegree centrality of the directed peer relationship network (Wassermann and Faust, 1994). For example, if mentee A has only peer relationships with mentees B and C, then the peer group size of mentee A is two. For each mentee, two values were derived from the adjacency matrices of peer relationships, indicating the number of peer relationships a mentee had during the first 4 weeks of mentoring and during the remaining time of mentoring.

#### Covariates

We also included several control variables that are important for our later analysis.

##### Age

The age (in years) of a mentee at the beginning of mentoring.

##### Mentoring group membership

As mentioned in section “The Online Mentoring Program CyberMentor as a Study Setting,” mentoring dyads share a “virtual room” with another mentoring dyad on the platform. We call this group of four individuals (or two mentoring dyads) mentoring group. Although every mentee theoretically can communicate with every other mentee or mentor in the program, this might not be the case in actuality. Through the design of the CyberMentor website, the mentee might be more aware of a partner mentee (and the mentor of the other dyad) in the mentoring group, theoretically increasing the probability of an exchange between two mentees of the same mentoring group. For this reason, we included mentoring group membership as a covariate.

##### Mentoring experience

After 1 year of mentoring, the mentees are offered the opportunity to participate in mentoring program for another year. Thus, our sample contained both mentees without previous mentoring experience (i.e., inexperienced, coded as “0”) and mentees with mentoring experience (i.e., experienced, coded as “1”). As mentees’ experience might have an influence on their mentoring outcomes, we controlled for mentoring experience in our analyses.

##### Mentor relationships

The number of mentors with which a mentee exchanges email and private chat messages during mentoring might affect the mentee’s mentoring outcome. We included the number of mentors a mentee had contact with during mentoring by using the same threshold of written emails and private chat messages for determining peer relationships (i.e., 4 written emails or 15 written private chat messages).

### Plan of Analysis

All statistical analyses were conducted within the R software environment for statistical computing and graphics (R Core Team, 2017) and the R package psych v1.8.12 (Revelle, 2018) unless otherwise stated. We mainly carried out four steps:

1. Treatment of missing values in our two variables of interest (i.e., confidence in own STEM abilities and STEM-related activities),
2. Pre-analysis, how the participants that we excluded from the analyses (mentees without at least one peer) differ from participants that were included in the analysis (mentees with at least one peer),
3. Descriptives regarding mentoring outcomes and the peer relationship network,
4. Longitudinal network analyses to test our four hypotheses concerning the development of mentees’ confidence in their own STEM abilities and their STEM-related activities.

For our longitudinal network analyses, we set our alpha level to 0.1. With an increased alpha of 0.1 instead of the conventional 0.05 the false negative rate is decreased (Miller and Ulrich, 2019). By conducting (to our knowledge) the first study examining peer influences in online mentoring, it was important for us to minimize false negative outcomes and thereby open up more room for potential future research (as suggested by Fiedler et al., 2012). For our other analyses (i.e., step 1–3), we used the (for social sciences) conventional alpha level of 0.05.

#### Missing Data

For imputing missing data, we utilized the R package MICE v2.30 and used the implemented predictive mean matching method for multiple imputation (van Buuren and Groothuis-Oudshoorn, 2011). We adjusted the number of imputations according to recommendations for the current missing pattern, i.e., 40 imputed datasets for 50% missing values, as recommended by Graham et al. (2007). We observed sufficient convergence of the algorithm (using 40 iterations).

The current version of the package for longitudinal network analysis, RSiena v1.2-4 (Ripley et al., 2018) cannot handle multiple imputed data (regarding the mentoring outcome variables, i.e., confidence in own STEM abilities and STEM-related activities). Thus, we combined the imputed data sets by calculating the mean of each 40 imputed values for each cell of the final data frame.

#### Pre-analysis: Differences Between Mentees With at Least One Peer Relationship and Mentees Without Any Peer Relationships

In our pre-analysis, we wanted to show, that the included mentees were fairly similar to the group of excluded mentees at the start of the mentoring. Moreover, if a difference between the two groups is detected after 6 months of mentoring, this would already be a (weak) indication that the exchange with peers can have an impact on the mentoring success of mentees. Thus, we compared the two groups with independent *t*-tests and the false discovery rate (FDR) correction for multiple comparisons (Benjamini and Hochberg, 1995) regarding their age, confidence in their own STEM abilities (T1 and T2), and their STEM-related activities (T1 and T2).

#### Descriptives

We derived descriptives of the mentoring outcomes (i.e., confidence in own STEM abilities and STEM-related activities) and peer relationships as well as network related measures, i.e., total number of existing relationships between mentees, the average degree centrality, density, and reciprocity. The average degree centrality corresponds to the average number of peers a mentee has during online mentoring. “Density” refers to the proportion of observed peer relationships (edges) relative to the – hypothetically – total number of possible peer relationships (edges); possible values can range between zero and one. The reciprocity in the peer relationship network represents the amount of peer relationships (edges) in the network that are reciprocal; possible values can range between zero and one.

#### Longitudinal Network Analysis

The main longitudinal social network analysis was done with the R package RSiena v1.2-4 (Ripley et al., 2018). The aim of the analysis is to explain the change of confidence in mentees’ own STEM abilities and their STEM-related activities during 6 months of mentoring. In this method, all four hypotheses are considered simultaneously (and thus, a possible confounding between them can be considered).

In Table 1 and Supplementary Table A1, all relevant parameters used in the model are described in detail. Table 1 describes the effects that are used to answer the research questions (i.e., the effects that determine the development of the mentoring outcomes, including possible confounding covariates). The simultaneous development of the associated peer relationship networks (including the control of selection effects) are described in Supplementary Table A2. In the following – for easier understanding of the results – the effect names used to test our hypotheses are listed: *Hypothesis 1:* Peer influence (Average similarity), *Hypothesis 2:* Moderation of mentee’s age on peer influence (Average similarity × ego’s age), *Hypothesis 3:* Influence of a mentee’s peer group size (Indegree), *Hypothesis 4:* Moderation of mentee’s age on influence of peer group size (Age × peer group size).

Note that all variables are centered internally by RSiena. This means that the values of interaction effects with age (i.e., hypothesis 2 and hypothesis 4) must be interpreted in the following way. A positive value of the moderation effect in hypothesis 2 would mean: mentees below average age become more dissimilar to their peers and mentees above average age become more similar to their peers (with regard to the mentoring outcome considered). A negative effect would mean that mentees below average age would become more similar to their peers and mentees above average age would become less similar. A more detailed explanation on how to interpret all effects, can be found in Table 1 and Supplementary Table A1.

As RSiena can be tweaked in many ways, after several test runs, we decided to increase the iteration steps from the initial four up to five, to increase the precision of the algorithm. To increase the estimation precision of the standard error (and thus the precision of the *p*-value), we increased the steps of the third phase of the RSiena estimation process to 4000 as recommended (Ripley et al., 2018). The participants’ mentoring outcome variables were negatively skewed; therefore, we decided to use the boundary-absorbing behavior model. As Ripley et al. (2018) state, it shows better fit, by allowing changing the mentoring outcome variable one step further even though the current state is already in its maximum value.

## Results

### Missing Data

An inspection of the data revealed that the missing values followed a missing at random pattern. In the online questionnaire dataset, there was a mean rate of 22% missing values at the first measurement point T1 and a mean rate of 50% missing values at the second measurement point T2. In order to impute missing values based on maximum information, we utilized multiple imputations with the complete (*N* = 430 cases) questionnaire data set (Newman, 2014). All subsequent analyses were then performed on the data obtained.

### Pre-analysis: Comparison Between Mentees With at Least One Peer Relationship and Mentees Without Any Peer Relationship

We compared the group of mentees that we included into our further analyses (mentees with at least one peer relationship; *N* = 124) with the group of mentees that we excluded from the analyses (mentees without a peer relationship; *N* = 306) with help of an independent *t*-tests, using FDR correction for multiple comparisons (Benjamini and Hochberg, 1995), regarding their age, confidence in their own STEM abilities, and their STEM-related activities. The results are shown in Table 2. At the beginning of mentoring (T1), the two groups did not differ significantly in age or regarding the two dependent variables: confidence in own STEM abilities and STEM-related activities.

**TABLE 2:** Results of the *t*-tests between mentees and without a peer relationship (wop) with at least one peer relationship (wp).

Variable	*M*_*wop*_ (SD)	*M*_*wp*_ (SD)	*t*(428)	*p*	*p*_*FDR*_	*d*
Age	14.31 (2.09)	14.31 (2.17)	0.02	0.99	0.99	0.00
Confidence in own STEM abilities T1	4.38 (0.97)	4.58 (0.91)	1.92	0.06	0.09	0.20
Confidence in own STEM abilities T2	4.38 (0.81)	4.57 (0.76)	2.31	0.02	0.05	0.25
STEM-related activities T1	3.72 (0.90)	3.89 (0.86)	1.77	0.08	0.10	0.19
STEM-related activities T2	3.85 (0.66)	4.06 (0.69)	2.96	< 0.01	**0.02**	0.31
Number of peer relationships T1	0 (0)	1.15 (1.75)	–	–	–	–
Number of peer relationships T2	0 (0)	1.58 (2.81)	–	–	–	–

**p*_*FDR*_ is the false positive rate corrected significance level; *d* is Cohen’s *d*. *p*_*corr*_ < 0.05 is marked bold.*

After 6 months of mentoring (T2), significant differences between the two groups indicate that mentees that interacted more intensely with other mentees on the platform had higher values in STEM-related activities than mentees that stayed relatively isolated from other mentees (*t*(428) = 2.96, *p*_*FDR*_ = 0.01, Cohen’s *d* = 0.31). Mentees with peer relationships showed similar confidence in their own STEM abilities after 6 months of mentoring at T2 compared to mentees without peer relationships (*t*(428) = 2.31, *p*_*FDR*_ = 0.05, Cohen’s *d* = 0.25).

### Descriptives

#### Mentoring Outcomes

The descriptives of the two mentoring outcomes: confidence in own STEM abilities and STEM-related activities, and the number of peer relationships of the mentees can be found in Table 2. Mentees did not differ significantly between T1 and T2 regarding their means in confidence in own STEM abilities (paired *t*-test, *t*(123) = −0.02, p_*FDR*_ = 0.98, Cohen’s | *d*| < 0.01) or STEM-related activities (*t*(123) = 2.26, p_*FDR*_ = 0.05, Cohen’s *d* = 0.22).

#### Peer Relationship Networks

The descriptive statistics of the two waves of the peer relationship network at the beginning (4 weeks) of the mentoring and the remaining 5 months of mentoring are shown in Table 3. Overall, the peer relationship network became denser throughout mentoring. The reciprocity remained unchanged, i.e., 83% of all peer relationships were reciprocal.

**TABLE 3:** Descriptive statistics of the used networks.

Network	Number of nodes	Number of edges	Average degree	Density	Reciprocity
Wave T1	124	143	1.15	0.009	0.83
Wave T2	124	196	1.58	0.013	0.83

### Results of Longitudinal Network Analyses

#### Model Convergences and Quality

To anticipate possible convergence problems, we examined the so-called Jaccard index. This is the amount of unchanged edges between T1 and T2 of the peer relationship networks, and the calculated value of 0.37 is considered good (Ripley et al., 2018).

For assessing convergence of the following analyses, the indices “convergence t ratios” and “overall maximum convergence ratio” are suitable. Indications of good convergence are convergence t ratios smaller than 0.1 and maximum convergence t ratios smaller than 0.25 (Ripley et al., 2018). In all calculated models, the corresponding indices were satisfactory (refer to Table 4 for exact values).

**TABLE 4:** RSiena model results of both mentoring outcomes.

	Confidence in own STEM abilities				STEM-related activities			
Effects	Effect value	SE	*t* stat	*p*	Effect value	SE	*t* stat	*p*
**Hypotheses related effects**								
*Peer influence (Average similarity) (H1)*	–5.13	4.07	–1.26	0.21	2.53	2.58	0.98	0.16
*Moderation of mentee’s age on peer influence (Average similarity* × *ego’s age) (H2)*	–1.52	1.10	–1.38	**0.08**	–1.44	0.97	–1.49	**0.07**
*Peer group size (Indegree) (H3)*	0.23	0.22	1.04	0.15	0.05	0.09	0.54	0.30
*Moderation of mentee’s age on influence of peer group size (Age* × *Indegree) (H4)*	–0.21	0.15	–1.39	**0.08**	0.01	0.04	0.17	0.43
**Mentoring outcome related effects**								
*Linear shape*	–0.26	0.24	–1.10	0.27	0.05	0.14	0.37	0.71
*Quadratic shape*	–0.29	0.13	–2.26	**0.02**	–0.16	0.05	–3.34	**< 0.01**
*Experience*	0.29	0.26	1.13	0.26	0.68	0.21	3.20	**< 0.01**
*Age*	0.16	0.14	1.12	0.26	–0.06	0.07	–0.86	0.39
*Mentor relationships*	0.11	0.11	1.07	0.28	0.07	0.05	1.33	0.20

*All convergence *t* ratios < 0.07, overall maximum convergence ratios < 0.13; *p*-values smaller than 0.1 are marked bold; each *p*-value is for two-sided tests, except the *p*-values of “moderation of mentee’s age on peer influence,” “peer group size,” and “moderation of mentee’s age on influence of peer group size.”*

Moreover, we considered three indicators for model fit: the indegree distributions, the out-degree distributions, and the mentoring outcomes distributions. The corresponding Monte Carlo Mahalanobis distance tests were calculated, where a good fit is indicated by a non-significant *p*-value. Each value (in-degree, out-degree, and mentoring outcomes distributions) was in the non-significant range (*p* > 0.05), thus indicating an acceptable fit of the models.

#### RSiena Model Results

All results of the RSiena model regarding the research questions can be found in Table 4 and will be described in more detail. To help interpret the results, it is important to know the average number of “decision opportunities” a mentee is given in the RSiena simulation. As noted in Supplementary Table A2, in our RSiena simulations regarding confidence in own STEM abilities, each mentee is given on average 5.55 (SD = 1.67) “decision opportunities” to change her confidence in STEM abilities by a value of 0.5. Regarding STEM-related activities, each mentee is given on average 5.33 (SD = 1.09) “decision opportunities” to change her STEM-related activities by a value of 0.5. This means that a mentee can (on average) increase or decrease her mentoring outcome by a maximum value of 5 × 0.5 = 2.5 (if, at each “decision opportunity” in the simulation, she prefers the same change in her mentoring outcome).

##### Hypothesis 1: Mentees are subject to peer influence in online mentoring, regarding (H1a) confidence in own STEM abilities and (H1b) STEM-related activities

*Confidence in own STEM abilities.* We did not find evidence indicating unmoderated peer influence on confidence in own STEM abilities (β = −5.13, SE = 4.07, two-sided *p* = 0.21, Table 4). Therefore, we reject Hypothesis H1a.

*STEM-related activities.* We did not find evidence indicating unmoderated peer influence on STEM-related activities (β = 2.53, SE = 2.58, two-sided *p* = 0.32, Table 4). Therefore, we reject Hypothesis H1b.

##### Hypothesis 2: The mentee’s age moderates the peer influence that a mentee is subject to in the following way: younger mentees are more susceptible to peer influence (than older mentees) in both (H2a) their confidence in STEM abilities and (H2b) their STEM-related activities

*Confidence in own STEM abilities.* We found significant evidence for the hypothesized moderating effect of age on peer influence on confidence in own STEM abilities (β = −1.52, SE = 1.10, one-sided *p* = 0.08). The negative value of the effect (Table 4) indicates that younger mentees (below the average age) have a positive similarity effect, i.e., they tend to grow more similar to the mentees they have a peer relationship with regarding confidence in own STEM abilities. More precisely: If a 11.2 years old mentee (*mean* age – 1.5 SD) has peer relationships to mentees with *higher* confidence in their own STEM abilities than herself, then the mentee has a 65% higher chance of *increasing* her confidence in her own STEM abilities (by a value of 0.5, in the event of a “decision opportunity”) than without these mentees (given all other parameters are constant). In other words: if we compare two identical 11.2-year-old mentees, mentee A and mentee B, where mentee A has peers (with a *higher* confidence in their own STEM abilities) but mentee B has none, then in the event of a “decision opportunity,” the probability for mentee A to *increase* her confidence in own STEM abilities (by a value of 0.5) is 1.65 times higher than it is for mentee B. Analogously, mentee A is 1.65 times more likely to *decrease* confidence in her own STEM abilities if her peers have *lower* confidence in their own STEM abilities.

This susceptibility to peer influence decreases with increasing age. In the case of older mentees (above the average), this effect reverses, i.e., their STEM confidence is increasingly moving away from the mentees they have a peer relationship with. More precisely: If a 17.5 years old mentee (*mean* age + 1.5 SD) has peer relationships to mentees with *higher* confidence in STEM abilities than herself, then the mentee has a 65% higher chance of *decreasing* her confidence in STEM abilities (by a value of 0.5, in the event of a “decision opportunity”) than without these mentees (given all other parameters are constant). In other words: if we again compare two identical 17.5-year-old mentees, mentee C and mentee D, where mentee C has peers (with a *higher* confidence in their own STEM abilities) but mentee D has no peers, in the event of a “decision opportunity,” the probability for mentee C to *decrease* confidence in her own STEM abilities (by a value of 0.5) is 1.65 times higher than it is for mentee D. Analogously, mentee C is 1.65 times more likely to *increase* confidence in her STEM abilities if her peers have *lower* confidence in their own STEM abilities.

Overall, the younger a mentee is, the more likely she adapts the level of her own confidence in STEM abilities to the level of her peers’ confidence in their own abilities. Thus, we accept Hypothesis H2a.

*STEM-related activities.* We found marginally significant evidence indicating the hypothesized moderating effect of age on peer influence on STEM-related activities (β = −1.44, SE = 0.97, one-sided *p* = 0.07). The negative value of the corresponding effect (Table 4) indicates, that younger mentees (below the average age) have a positive similarity effect, i.e., they tend to become more similar to the mentees they have a peer relationship in regards to STEM-related activities. More precisely: If a 11.2 years old mentee (*mean* age – 1.5 SD) has peer relationships with mentees with *higher* STEM-related activities than herself, then the mentee has a 61% higher chance to *increase* her STEM-related activities (by a value of 0.5, in the event of a “decision opportunity”) than without these mentees (given all other parameters are constant). In other words: if we compare two identical 11.2-year-old mentees, mentee A and mentee B, where mentee A has peers (with a *higher* level of STEM-related activities) but mentee B has none, then in the event of a “decision opportunity,” the probability for mentee A to *increase* her STEM-related activities (by a value of 0.5) is 1.61 times higher than it is for mentee B. Analogously, mentee A is 1.61 times more likely to *decrease* her STEM-related activities if her peers have *lower* STEM-related activities.

This susceptibility to peer influence decreases with increasing age. In the case of older mentees (above the average), the effect reverses, i.e., their level of STEM-related activities is increasingly moving away from the mentees they have a peer relationship with. More precisely: If a 17.5 years old mentee (*mean* age + 1.5 SD) has peer relationships to mentees with *higher* STEM-related activities than herself, then the mentee has a 61% higher chance to *decrease* her STEM-related activities (by a value of 0.5, in the event of a “decision opportunity”) than without these mentees (given all other parameters are constant). In other words: if we again compare two identical 17.5-year-old mentees, mentee C and mentee D, where mentee C has peers (with a *higher* degree of STEM-related activities) but mentee D has none, then in the event of a “decision opportunity,” the probability for mentee C to *decrease* her STEM-related activities (by a value of 0.5) is 1.61 times higher than it is for mentee D. Analogously, mentee C is 1.61 times more likely to *increase* her STEM-related activities if her peers have *lower* STEM-related activities.

Overall, the younger a mentee is, the more likely she adapts her own level of STEM-related activities to the level of her peers’ STEM-related activities. Thus, we accept Hypothesis H2b.

##### Hypothesis 3: The size of the peer group (operationalized by the number of peers that regularly send messages to the mentee) contributes positively to the mentoring outcomes in the following way: The larger the size of the peer group a mentee interacts with the more positive the development of her (H3a) confidence in own STEM abilities and (H3b) STEM-related activities

*Confidence in own STEM abilities.* We found no evidence that the size of a mentee’s peer group positively contributes to mentees’ confidence in own STEM abilities (β = 0.23, SE = 0.22, one-sided *p* = 0.15, Table 4. Thus, we reject hypothesis H3a.

*STEM-related activities.* We found no evidence that the size of a mentee’s peer group positively contributes to STEM-related activities (β = 0.05, SE = 0.09, one-sided *p* = 0.30, Table 4). Thus, we reject hypothesis H3b.

##### Hypothesis 4: The influence of the size of the peer group (operationalized by the number of peers that regularly send messages to the mentee) on the mentoring outcomes is moderated by the mentee’s age in the following way: The older mentees are, the less they benefit from an increasing size of their peer group regarding (H4a) their confidence in STEM abilities and (H4b) their STEM-related activities

*Confidence in own STEM abilities.* We found significant evidence of a moderation of age on the influence of the size of the peer group on the confidence in own STEM ability (β = −0.21, SE = 0.15, one-sided *p* = 0.08). The expected negative value of the effect (Table 4) indicates, that for younger mentees (below the average age) each additional peer relationship increases the likelihood that the mentee improves her confidence in own STEM abilities, exponentially (i.e., exp[(−1.5×*SD*_age_) × (−0.21) × *n*], where *n* stands for the number of peers, *SD*_age_ for the standard deviation of mentees’ age, and exp for the exponential function). More precisely, one additional peer relationship of an 11.2 years old mentee (*mean* age – 1.5 SD) increases the mentee’s likelihood by 100% of increasing her level of confidence in own STEM abilities (by a value of 0.5, in the event of a “decision opportunity”; given all other parameters are constant). Four additional peer relationships of an 11.2 years old mentee increase the aforementioned likelihood by 1499% (i.e., nearly 15 times). In other words: if we compare two identical 11.2-year-old mentees, mentee A and mentee B, where mentee A has four peers but mentee B has zero peers, then in the event of a “decision opportunity,” the probability for mentee A to *increase* her confidence in own STEM abilities (by a value of 0.5) is 15.99 times higher than for mentee B.

The observed moderation of mentee’s age indicates, that for the average old mentees, there is no peer group effect. Moreover, in older mentees, one additional peer relationship of a 17.5 years old mentee (*mean* age + 1.5 SD) increases the likelihood by 100% of a *decrease* in her level of confidence in own STEM abilities (analogous to young mentees, by a value of 0.5, in the event of a “decision opportunity”; given all other parameters are constant). Thus, we accept hypothesis H4a.

*STEM-related activities.* We found no evidence that the contribution of a mentee’s peer group to STEM-related activities is moderated by age (β = 0.01, SE = 0.04, one-sided *p* = 0.43, Table 4). Thus, we reject hypothesis H4b.

## Discussion

The main objective of our study was to investigate peer influence in online mentoring. We analyzed whether mentoring outcomes – namely confidence in own STEM abilities and STEM-related activities – are influenced by networking with other mentees on the mentoring platform. As research shows that peer influence differs during development (e.g., Steinberg and Monahan, 2007), we also investigated the moderating role of age for peer influence on mentoring outcomes. Furthermore, we investigated the role of peer group size concerning peer influence on mentoring outcomes (as indicated by e.g., Wang et al., 2016). To obtain more reliable estimates of peer influence effects, we controlled for selection processes that determine the peer relationship network evolution, for mentoring experience, as well as for the number of mentors in a mentee’s email and private chat message exchange. As our method, we conducted a longitudinal social network analysis, using an stochastic actor based simulation approach (Snijders et al., 2010; i.e., RSiena; Ripley et al., 2018).

Overall, our findings suggest peer influence for both our examined mentoring outcomes, i.e., confidence in own STEM abilities and STEM-related activities in STEM. However, while we did not find an age-independent peer influence on mentoring outcomes, we found an age-moderated effect. Younger mentees tend to adapt to their peers’ average level of mentoring outcomes, whereas older mentees tend to distance themselves from the average mentoring outcome level of their peers.

This finding is consistent with research from offline contexts (Steinberg and Monahan, 2007; Aral and Walker, 2012) and online contexts outside the field of mentoring (Aral and Walker, 2012; Bapna and Umyarov, 2015). Some researchers would call this pattern of results increasing resistance against peer influence (Steinberg and Monahan, 2007). Our results indicate that resistance against peer influence in our sample seems to manifest itself in the following way: older mentees (i.e., girls above the mean age of 14.3 years) become more dissimilar to their peers, both in their confidence in own STEM abilities and their STEM-related activities.

The observed results might be attributable to the way peer relationships between mentees are formed. For example, our peer network development statistics (see Supplementary Table A2) show that relationships between young, inexperienced mentees and older, more experienced mentees are more likely to develop. Thus, younger mentees might have a type of unofficial “peer-mentor” that is more similar to them than their official mentor (Colvin and Ashman, 2010), and by whom they are more strongly influenced (Karcher, 2013). The same mechanism does not ring true for older mentees. In future studies this assumption – and especially potentially existing informal peer mentoring relationships in online mentoring – should be considered, preferably with a bigger sample. For example, in future studies, mentees could be explicitly asked if and with which of their peers a form of mentoring relationship exists.

Another age-dependent finding of our study was that the size of the peer group positively impacts confidence in own STEM abilities – but not STEM-related activities. The effect of peer group size on mentoring outcomes is higher in young mentees than in older mentees. More precisely: the larger the peer group of a young mentee is, the more likely she is to increase her confidence in own STEM abilities during mentoring. One reason for the supporting role of the size of the online peer group for young mentees might be that in comparison to other settings (e.g., school), during online mentoring mentees have the chance to communicate with other like-minded peers. This might lead mentees to re-evaluate themselves positively due to their newly enriched social environment (Berndt and Ladd, 1989), thus increasing their confidence in own STEM abilities. Moreover, a larger peer network seems to heighten the commitment to visit the online mentoring platform more often (Schimke et al., 2009), which in turn leads to positive mentoring outcomes (Stoeger et al., 2016). Older mentees (over 14.3 years of age), however, do not benefit from a large peer group size. The phenomenon of social comparison might explain our age moderated findings. Individuals tend to evaluate their own abilities, opinions, attitudes and other self-aspects in relation to other individuals (Guyer and Vaughan-Johnston, 2018). A comparison with status-higher individuals (upward comparison) often hinders self-aspects, whereas a comparison with status-lower individuals (downward comparison) tends to support self-aspects. Female students tend to have a tendency to compare upward (Pulford et al., 2018), an approach that increases with age (Martin and Kennedy, 2003), thus being in line with our observed results.

Surprisingly, we did not find any impact of the size of the peer group on STEM-related activities – neither age independent, nor age dependent. This finding might be put in perspective by a comparison with results of other studies on online mentoring (Hopp et al., 2014; Stoeger et al., 2016). First, it could be shown that STEM-related activities are influenced by STEM-related messages exchanged between mentees as well as between mentees and mentors (Stoeger et al., 2016). Here lie the main differences in our study since we only examined the messages between mentees and did not examine the message contents (STEM-related vs. non-STEM-related). Moreover, the impact of the peer group can be understood as a kind of contagion process (Dasgupta, 2011). Thus, it is important that the actual “virus” (i.e., embedded in STEM-related content) is exchanged in order to get infected. Earlier analyses showed that a network measure similar to the size of the peer group (i.e., Hopp et al., 2014) can have a positive influence on STEM-related activities of mentees, but only when the STEM content of communication within the network (i.e., STEM-related emails) was taken into account.

Furthermore, the mentors – and not the peers – of the mentees could be the main initiators of STEM-related activities. This assumption is supported by the implementation of mentoring in CyberMentor (i.e., the program under investigation). The program offers STEM-related project ideas (e.g., explaining everyday STEM-related phenomena or STEM experiment instructions) that are intended to strengthen the cooperation between mentor and mentee as well as the mentee’s STEM related activities. Several studies show that the program has a positive influence on mentees’ STEM activities (Stoeger et al., 2013, 2016). There is also evidence that mentors play an important role for increases in STEM activities (Stoeger et al., 2019). In future research, it would be interesting to investigate more thoroughly how peer- and mentor-influences interact when it comes to increasing STEM activities in the CyberMentor program.

### Limitations

In our study, we found initial evidence of peer influence on mentoring outcomes. However, there were several limitations to our research that should be kept in mind when interpreting the results.

First, we must address a few issues regarding our sample. Our sample consists exclusively of girls, which seems to be adequate for mentoring in STEM as programs in this area try to reduce negative gender-related stereotypes toward the field – something more easily achieved by exclusively providing female role models (e.g., Stout et al., 2011). However, it is not clear whether our results can be generalized to peer influence in STEM mentoring programs with male and female mentees. In these programs, peer influence might be moderated by gender, as suggested by some research (e.g., Brechwald and Prinstein, 2011). Moreover, to acquire more robust results of the used longitudinal network analysis method, our sample is based on active mentees, i.e., mentees that wrote at least four emails to another mentee over the course of 6 months. Thus, the results may not be generalizable to all participating mentees of CyberMentor. Overall, future studies should include a more varied sample to address the mentioned sample limitations.

Second, we found relatively weak effects, or in some cases no effects at all. One reason might be that – as earlier studies showed (Stoeger et al., 2016) – mostly girls with a high STEM interest are attending CyberMentor. This might lead to a restriction of variances and thus, to smaller effects. Another reason for the small effects could be the missing focus on the content of communication. Further research should include various aspects of the content of communication (e.g., relation to STEM or emotional characteristics) in order to better understand peer influence and relationships between mentees. The quality of relationships between mentees, but also between mentees and mentors might moderate peer influence. For example, mentees with a need for improvement in their relationship with their mentor might be more receptive to peer influence. However, it might also be the case (as one reviewer suggested) that individuals with stronger relationships with their mentor might be more likely to reach out to more peers (for example, because individuals with stronger mentor relationships might develop the confidence to do so). In future studies, the relationship quality between mentee and mentor should be taken into account. Overall, future studies should investigate further moderation effects on peer influence and use bigger sample sizes.

### Conclusion and Key Implications

Overall, and to the best of our knowledge, this is the first study that analyzed peer influence in online mentoring on mentoring outcomes for girls in STEM (i.e., confidence in own STEM abilities and STEM-related activities). Our study suggests that not only do mentors influence mentoring outcomes, peers (other mentees) with whom the girls communicate on the mentoring platform do, too. The age of the mentees seems to play an important role with younger mentees adapting to the mentoring outcome level of their peers over the course of mentoring, and with older mentees diverging from their peers over time. In addition, stronger networking with peers (i.e., the size of the peer group) on the platform increased confidence in own STEM abilities of the mentees in our study, but only for young mentees.

It is much too early to infer recommendations for practice from our results. Should future research support our findings there might be some suggestions for platform structuring and group composition in online mentoring for girls. Our results are initial evidence that the more peers a young mentee has, the better it might be for her confidence in own STEM abilities. Therefore, the development of peer relationships in online mentoring programs should be encouraged – especially for young mentees. To accomplish this, easily accessible communication possibilities can be offered on the online platforms. This might increase the probability that these means of communication will be used, which is also indicated by our results (see Supplementary Table A2): mentees communicate with peer mentees from their own mentoring group – with whom they share special communication tools – rather than with other mentees on the platform.

In addition, initial – albeit, highly speculative – we found indications for the set-up of mentoring groups. On the one hand, our results indicate that age is a moderator on peer group size (see Supplementary Table A2), i.e., older mentees are more likely to side with younger – and thus often inexperienced – mentees. On the other hand, we observed a moderation of the mentee’s age on peer influences in both mentoring outcomes (i.e., confidence in own STEM abilities and STEM-related activities): young mentees show a tendency to adapt the mentoring outcome of their peers, whereas older mentees show an opposite behavior. Taken together, a mentoring group where a young mentee has the possibility to exchange with an older, more experienced mentee might be beneficial for both parties. However, the evidence that the current study provides is too weak to make strong inferences regarding platform structuring and group composition in online mentoring for girls.

Overall, our results provide initial evidence of the positive impact of peer influence and networking between mentees in online mentoring for girls in STEM. From this, we derive tentative indications of beneficial mentoring group compositions. However, the limitations of this study should be considered. Further studies replicating and broadening the results are needed.

## Data Availability Statement

The datasets analyzed for this study are not available due to reasons of data protection. Requests to access the datasets should be directed to MH, manuel.hopp@fau.de.

## Ethics Statement

Ethical review and approval was not required for the study on human participants in accordance with the local legislation and institutional requirements. Written informed consent to participate in this study was provided by the participants’ legal guardian/next of kin.

## Author Contributions

MH conceived the original idea, processed all the data, conducted all the statistical analyses, and wrote the manuscript. HS revised the manuscript. AZ and HS provided feedback and supervised the work. All authors reviewed the final manuscript.

## Conflict of Interest

The authors declare that the research was conducted in the absence of any commercial or financial relationships that could be construed as a potential conflict of interest.

## Footnotes

1 At the beginning of mentoring, active mentees did not differ significantly in their age or in regard to the two mentoring outcomes. However, after 6 months of mentoring, slightly better mentoring outcomes were found for the mentees with at least one peer relationship. A more detailed comparison of the included and excluded mentees can be found at the beginning of the results section. In the following, we will only refer to the sample consisting of 124 active mentees.
