# Supplementary material for: The Supporting Role of Mentees’ Peers in Online Mentoring: A Longitudinal Social Network Analysis of Peer Influence
Source: Front Psychol. 2020 Aug 14;11:1929. doi: 10.3389/fpsyg.2020.01929 (PMC7456988; doi:10.3389/fpsyg.2020.01929)
Supplement: Supplementary file 1 [file Data_Sheet_1.PDF]

Supplementary Material

Appendix

Table A1

Short Description of Included Effects for the Simulation of the Peer Relationship Networks in Our RSiena Models.

Effects	Short description
**Peer relationship network related effects**	
Reciprocity	Social networks show almost always positive reciprocity tendencies (for more details, see Squartini et al., 2013), meaning, if person A nominates person B as a friend at time 1 (and keeps doing that), then person B tends to nominate person A as a friend at a later point in time. A positive value indicates this tendency.
Triadic closure (GWESP)	In the dynamics of friendship or social relationship formation, an individual befriends more likely the friends of his or her friends, this phenomenon is known as triadic closure (Rapoport, 1953). GWESP stands for geometrically weighted edgewise shared partners and is a measurement for triadic closure (Hunter, 2007; Snijders et al., 2006). A positive value indicates such a tendency.
Matthew effect of popularity (Indegree × Indegree)	A positive value represents the tendency, that a mentee with many (received) peer relationships at T1 (i.e. being “popular”) tend to get even more (received) peer relationships because of that (i.e. get more “popular”).
Anti-isolate	This is contact-seeking behavior. A positive value indicates such a tendency. We included this effect solely to achieve better network statistics fits.
Communication possibilities<br><br>*(Same Mentoring-Group)*	On CyberMentor, being a member of the same Mentoring-Group corresponds to a higher probability of communicating with each other. A positive value would indicate this.
Similarity in age	In social networks, homophily can be found concerning age, which means that mentees of a similar age are more likely to establish a peer relationship. A positive value indicates that tendency.
Ego age	Control for the age similarity effect. A positive value means that older mentees are more likely to build peer relationships than younger mentees.

Alter age	Control for the age similarity effect. A positive value means that older mentees are more likely to be nominated as peers than younger mentees.
Similarity in mentoring outcomes	To control influence effects for possible selection effects, we included effects for similarity of the corresponding mentoring outcome variable. If this effect has a positive value, it means that a peer relationship is more likely to develop between two mentees, if they have a similar level in the mentoring outcome at T1.
Ego mentoring outcomes	Control for the mentoring outcome similarity effect. A positive value means that mentees with a higher mentoring outcome level at T1 are more likely to build peer relationships than mentees with a low level.
Alter mentoring outcomes	Control for the mentoring outcome similarity effect. A positive value means that mentees with a higher mentoring outcome level at T1 are more likely to receive peer relationships than mentees with a low level.
**Additional simulation specific effects**	
Rate	In the simulation of RSiena, each actor (i.e. mentee) receives several decision points in which she can either establish/resolve peer relationships or change her mentoring outcome. This rate parameter gives information about the number of changes regarding her peer relationship network or mentoring outcome.
Outdegree	This effect is important to control for the density in the network. As Ripley and colleagues (2018) write in the manual for RSiena, the specific value of the density parameter is not very important but should always be included.

Table A2

RSiena Network Evolution Model Results of Both Mentoring Outcomes

Effects	Peer relationship network estimation of confidence in own STEM ability analysis				Peer relationship network estimation of STEM related activities analysis			
	Effect Value	*S.E.*	t stat	*p*	Effect Value	*S.E.*	t stat	*p*
**Peer relationship network related effects**								
Reciprocity	7.83	1.13	6.88	<**.001**	8.79	1.39	6.31	**<.001**
Triadic closure (GWESP)	1.96	0.62	3.16	**.002**	2.38	0.76	3.12	**.002**
Matthew effect of popularity (Indegree × Indegree)	1.61	0.47	3.40	<**.001**	1.74	0.58	3.01	**.002**
Anti-isolate	4.05	1.33	3.06	**.002**	5.42	1.87	2.89	**.004**
Communication possibilities (Same Mentoring-Group)	4.80	0.59	8.09	<**.001**	4.88	0.86	5.69	**<.001**
Similarity in age	2.89	0.91	3.19	**.001**	2.94	0.90	3.29	**.001**
Ego age	-0.16	0.15	-1.15	.249	-0.27	0.12	-2.20	**.028**
Alter age	0.30	0.15	2.09	**.037**	0.33	0.16	2.09	**.036**
Similarity mentoring outcome	-0.56	2.03	-0.27	.784	-0.21	1.68	-0.12	.903
Ego mentoring outcome	0.55	0.26	2.12	**.034**	-0.15	0.28	-0.56	.578
Alter mentoring outcome	-0.02	0.30	-0.07	.943	0.15	0.32	0.47	.640
**Additional simulation specific effects**								

Rate network	5.30	0.96	-	-	5.48	0.99	-	-
Rate mentoring outcome	5.55	1.67	-	-	5.33	1.09	-	-
Outdegree	-9.67	1.47	-6.56	<**.001**	-10.54	1.87	-5.63	**<.001**

Note: all convergence *t* ratios <.07, overall maximum convergence ratio <.13; *p* values smaller than .1 are marked bold; each *p* value is for a two-sided test.
